# Molecular alterations and targeted therapy in pancreatic ductal adenocarcinoma

**DOI:** 10.1186/s13045-020-00958-3

**Published:** 2020-10-02

**Authors:** Yunzhen Qian, Yitao Gong, Zhiyao Fan, Guopei Luo, Qiuyi Huang, Shengming Deng, He Cheng, Kaizhou Jin, Quanxing Ni, Xianjun Yu, Chen Liu

**Affiliations:** 1 Department of Pancreatic Surgery, Fudan University Shanghai Cancer Center, NO.270 DongAn Road, Shanghai, 200032 China; 2 Department of Oncology, Shanghai Medical College, Fudan University, Shanghai, 200032 China; 3 Shanghai Pancreatic Cancer Institute, Shanghai, 200032 China; 4 Pancreatic Cancer Institute, Fudan University, Shanghai, 200032 China

**Keywords:** Therapeutic targets, Precision oncology, Pancreatic ductal adenocarcinoma, Oncogenes, Tumour suppressors, Epigenetics, Synthetic lethality, Immunotherapy

## Abstract

Pancreatic ductal adenocarcinoma (PDAC) is a malignancy characterized by a poor prognosis and high mortality rate. Genetic mutations and altered molecular pathways serve as targets in precise therapy. Using next-generation sequencing (NGS), these aberrant alterations can be identified and used to develop strategies that will selectively kill cancerous cells in patients with PDAC. The realization of targeted therapies in patients with PDAC may be summarized by three approaches. First, because oncogenes play a pivotal role in tumorigenesis, inhibition of dysregulated oncogenes is a promising method (Table 3). Numerous researchers are developing strategies to target oncogenes, such as KRAS, NRG1, and NTRK and related molecules, although most of the results are unsatisfactory. Accordingly, emerging strategies are being developed to target these oncogenes, including simultaneously inhibiting multiple molecules or pathways, modification of mutant residues by small molecules, and RNA interference. Second, researchers have attempted to reactivate inactivated tumour suppressors or modulate related molecules. TP53, CDKN2A and SMAD4 are three major tumour suppressors involved in PDAC. Advances have been achieved in clinical and preclinical trials of therapies targeting these three genes, and further investigations are warranted. The TGF-β-SMAD4 signalling pathway plays a dual role in PDAC tumorigenesis and participates in mediating tumour-stroma crosstalk and modulating the tumour microenvironment (TME); thus, molecular subtyping of pancreatic cancer according to the SMAD4 mutation status may be a promising precision oncology technique. Finally, genes such as KDM6A and BRCA have vital roles in maintaining the structural stability and physiological functions of normal chromosomes and are deficient in some patients with PDAC, thus serving as potential targets for correcting these deficiencies and precisely killing these aberrant tumour cells. Recent clinical trials, such as the POLO (Pancreas Cancer Olaparib Ongoing) trial, have reported encouraging outcomes. In addition to genetic event-guided treatment, immunotherapies such as chimeric antigen receptor T cells (CAR-T), antibody-drug conjugates, and immune checkpoint inhibitors also exhibit the potential to target tumours precisely, although the clinical value of immunotherapies as treatments for PDAC is still limited. In this review, we focus on recent preclinical and clinical advances in therapies targeting aberrant genes and pathways and predict the future trend of precision oncology for PDAC.

## Background

Pancreatic cancer is a well-known lethal disease with similar mortality and morbidity rates. Its incidence continues to increase, while its 5-year relative survival rate remains the lowest (9%) [1] among all cancers. Furthermore, most patients with pancreatic cancer experience recurrence and metastasis, even after curative resection. Despite advances in surgical approaches and the emergence of various chemotherapy regimens, its poor prognosis has not improved in the last several decades. Studies exploring new therapeutic methods are urgently needed.

Targeted therapy highlights the association between neoplastic characterization and individual therapeutic responses. It is based on genomics and biomarker expression, suggesting that genomic mutations along with their altered downstream pathways are potentially useful pharmacological targets or prognostic indicators. Advances in genome sequencing have enabled researchers to rapidly identify the genetic differences between tumour cells and normal cells [2].

Currently, many other types of tumours, such as breast and ovarian cancers, are treated in a precise manner. However, the only precise therapeutic agent approved for pancreatic ductal adenocarcinoma (PDAC) is erlotinib, which only slightly prolongs survival [3, 4]. Precision oncology is also expected to be applied to PDAC to increase therapeutic efficacy and reduce toxicity, hence facilitating more cost-effective medicine. In this review, we summarize recent advances in targeted therapy for PDAC.

## Role of next-generation sequencing (NGS) in targeted therapy

### Screening and typing patients with PDAC

Advanced technologies facilitate the diagnosis of PDAC and the detection of tumour mutations. In addition to tumour biopsies, NGS has been performed using multiple types of specimens, such as pancreatic cyst fluid [5], secretin-stimulated juice [6], and cell-free DNA collected from the blood [7]. The use of more easily acquired specimens not only facilitates PDAC screening [8] but also obviates complications and costs.

Whole-genome sequencing reveals the mutational landscape of PDAC, and PDAC has been divided into four subtypes according to the variations in chromosomal structure: stable, locally rearranged, scattered, and unstable, each of which has its own distinctive mutational signatures [9, 10]. Researchers have also attempted to combine transcriptomic and genomic analysis to define PDAC subtypes because the mutational and transcriptional profiles do not overlap and an integrated genomic and transcriptomic analysis may reveal PDAC heterogeneity more thoroughly [11, 12].

The categorization of PDAC into various subtypes has potential clinical applications, as the basis of precision oncology is differentiating patients who may respond to a certain treatment from others and recognizing promising therapeutic targets [13]. Inspiringly, The Know Your Tumour programme revealed that 26% of the PDAC profiles harboured actionable molecular alterations, and molecularly matched precise therapy for patients with PDAC substantially improved their overall survival (OS) (hazard ratio (HR) = 0.42, *P* value = 0.0004) [14].

### Detecting early mutations and guiding targeted therapy

Tumorigenesis mainly results from genetic aberrations [15, 16]. As the amount of information about the genetic events involved in PDAC increases, the identification of ideal therapeutic targets is becoming possible. The aberrant genetic events in PDAC are generally divided into oncogene activation and tumour suppressor inactivation, and the four major genetic mutations observed in PDAC occur in KRAS, TP53, CDKN2A and SMAD4. These four commonly mutated major genes have been used to characterize PDAC and provided a pleiotropic roadmap for identifying ideal targets that may benefit most patients [17]. PDAC develops through a stepwise progression, and the progression from preneoplastic lesions to PDAC is a process characterized by the accumulation of genetic mutations. Early-stage precancerous lesions already appear to harbour mutations that are required for PDAC progression [18, 19]. For example, the most common KRAS and TP53 mutations are detected in early-stage intraepithelial neoplasia [20], suggesting that they play an important role in tumour onset.

In addition to the four major canonical genes involved in PDAC, genes involved in stabilizing chromatin, remodelling chromatin or editing point mutations in cancer cells, e.g. BRCA, APOBEC and KDM6A, also warrant investigation. Their low mutation frequencies in PDAC raise doubt about their clinical importance. Nonetheless, the poor prognosis of patients with PDAC suggests that any target, even if few people benefit from a treatment targeting that gene, is encouraging and merits investigation. Based on the aforementioned genetic events, researchers have attempted to therapeutically target these genetic variants and the altered pathways. In general, targeted treatment has been implemented using three approaches: inhibiting the dysregulated activation of oncogenes, interfering with the inactivation of tumour suppressors and exploiting the biological functional deficiency of certain genes, such as BRCA. Recent genetic-based explorations of precise targets in PDAC are shown in Table 1.

**Table 1:** Potential therapeutic targets of altered genes and aberrant pathways in PDAC

Gene alterations (Targets)	Mutation rate	Potential target	Therapeutic mechanism	Promising agents	Combination partner	Study phase	Reference
KRAS	90	EGFR	Target inhibition	Erlotinib	Gemcitabine	Phase III	CONKO-005
				Afatinib	Capecitabine	Phase I	NCT02451553
				Nimotuzumab	Gemcitabine	Phase II	OSAG101-PCS07, NCT00561990, EudraCT 2007-000338-38
			Combined inhibition	Erlotinib	Selumetinib	Phase II	NCT01222689
			Nanoparticle-based delivery	C18-EEG-GE11	Olaparib Gemcitabine	Mouse model	2018, American Chemical Society
		KRAS G12D/G12V	RNA interference or gene ablation	*siG12D-LODER*^*TM*^	Gemcitabine	Phase I/IIa	NCT01188785
		KRAS G12C	Cysteine residue modification	MRTX849	Afatinib Pembrolizumab Cetuximab	Phase I/II	NCT03785249, NCT04330664
		MEK	Multiple pathway inhibition (MEK inhibitors as backbone)	Trametinib	ABT-263 (Navitoclax, BCL-XL inhibitor)	Xenografts	2013, Cancer Cell
				AZD6244 (Selumetinib)	BKM120 (Buparlisib, PI3K inhibitor)	Mouse model	2014, Clinical Cancer Research
			Synthetic lethality	Trametinib	SHP099(SHP2 inhibitor)	Mouse model	2019, Molecular Cancer Therapeutics
					SHOC2 knock out		2019, Cell Reports
			Exploitation of EMT	Trametinib	Rosiglitazone		Certified in other epithelial cancer
			Immunosuppressive TME modulation	GDC-0623 (Cobimetinib)	CD40 antibody	Mouse model	2020, Nature Communication
				Trametinib	Palbociclib and PD-L1 antibody	Mouse model	2020, Gut
		PI3K	Pathway Inhibition	Rigosertib		Phase II/III	NCT01360853
			Multiple pathway inhibition	MK-2206	Selumetinib	Phase II	2017, JAMA of Oncology NCT01658943
				GDC-0941 (Pictilisib)	Ulixertinib	Cancer cell lines	2018, Molecular Cancer Therapeutics
TP53	70	P53	Missense mutant P53 reactivation	APR-246 (Cysteine binding compound)			Ongoing trials in other malignancies
				COTI-2 (Zinc chelating compound)	Cisplatin	Phase I	NCT02433626
		MDM2	Target inhibition	Nutin MA242		Mouse model	2018, Cancer Research
CDKN2A	60	CDK4/6	Cell cycle arrest	Palbociclib	Ulixertinib	Phase I	NCT03454035
				Ribociclib	Trametinib	Phase I/II	NCT02703571
				Abemaciclib		Phase II	NCT02981342
SMAD4	50	TGFβ	Pathway inhibition	Galunisertib	EcN	Mouse model	2019, Theranostics
					Gemcitabine	Phase I/II	NCT01373164
KDM6A	20	KDM6A	MYC upregulation reversion	JQ1 (BET inhibitor)		Mouse model	2018, Cancer Cell
			H3K27 methylation prevention	GSK126 (EZH2 inhibitor)		Cancer cell lines	2018, Nature Medicine
BRCA	5	PARP	Synthetic lethality	Olaparib		Phase III	POLO trial, NCT02184195
MSI-H/dMMR	1	PD-1	Immune checkpoint blockade	Pembrolizumab		Phase II	KEYNOTE-158, NCT02628067
NRG	0.5	ERBB3	Target inhibition	MCLA-128 (zenocutuzumab)		Phase I/II trials	NCT02912949
NTRK	0.3	TRK	TRK inhibition	Larotrectinib Entrectinib		Pooled analysis of phase I/II trials	2019/2020, Lancet Oncology
			NTRK mutations inhibition	Selitrectinib Repotrectinib		Phase I/II trials	NCT03215511 NCT03093116

*PDAC* pancreatic ductal adenocarcinoma; *BET* Bromodomain and extra-terminal domain; *TME* tumour microenvironment; *EMT* epithelial-mesenchymal transition; *ZSH* zeste homolog; *MSI-H* microsatellite instability-high; *PD-1* Programmed cell death protein 1; *dMMR* mismatch repair deficiency; *TRK* tropomyosin receptor kinase; *EcN* Escherichia coli strain Nissle 1917

## Oncogenes in PDAC and potential targets

### Oncogenic KRAS is responsible for tumorigenesis in most patients with PDAC

The most well-known oncogene involved in PDAC is RAS. RAS plays important roles in the signalling pathways regulating cell growth and differentiation to promote cell proliferation and differentiation and inhibit apoptosis. RAS switches between the inactive GDP-bound state and the active GTP-bound state, and recruited RAS guanine nucleotide exchange factors [21] and GTPase-activating proteins [22] are responsible for managing the transient activation of RAS.

**Fig 1:**
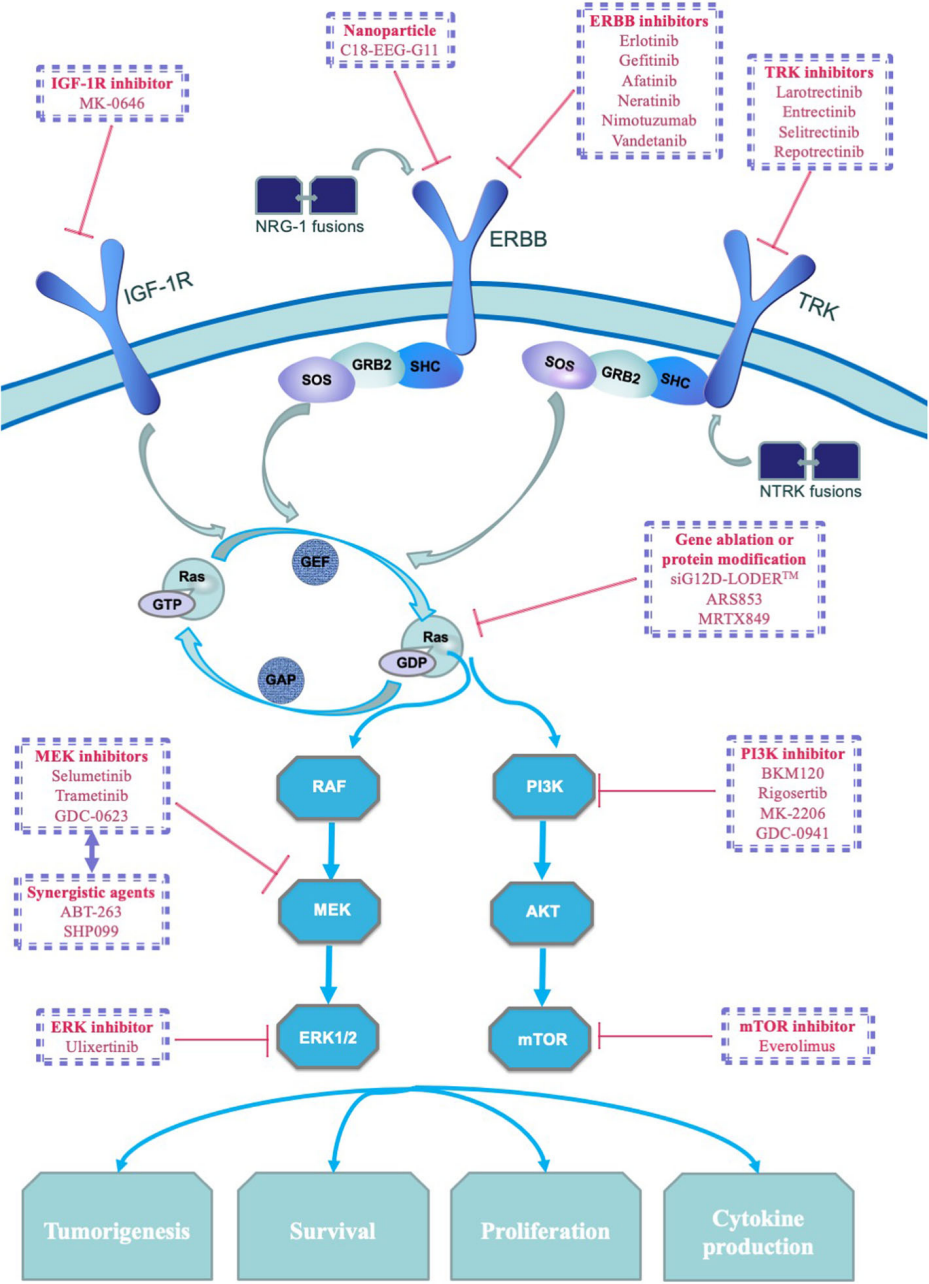
ERBB family comprises four receptor tyrosine kinases including the epidermal growth factor receptor (EGFR). Activation of EGFR recruits RAS guanine nucleotide exchange factors (GEFs) such as son-of-sevenless (SOS). GEFs and GTPase activating proteins (GAPs) switch RAS between the GTP-bound and GDP-bound states. The constitutive GDP-bound state activates multiple downstream molecules in PDAC. Gene fusions such as NRG1 fusions can also initiate PDAC via ectopic ERBB receptor signalling pathway. IGF-1R has crosstalk with EGFR and produces tumour resistance to EGFR inhibitors. Various inhibitors could inhibit RAS signalling pathway molecules by targeting corresponding molecules such as EGFR, MEK, PI3K

KRAS mutations are the most common mutations identified in human solid tumours, and approximately 90% of patients with PDAC harbour the G12 mutation in KRAS [23–26]. The most frequent point mutations at G12, G13 and Q61 [22] inhibit the intrinsic GTPase activity of RAS, thus sustaining the GTP-bound state of the RAS protein, which is established to be oncogenic [27, 28] (Fig. 1). Constitutively activated KRAS subsequently upregulates the endogenous expression of the upstream protein epidermal growth factor receptor (EGFR) and induces its hyperactivation [29, 30], and increased RAS levels and EGFR activity induce robust increases MEK/ERK activity, leading to intraepithelial neoplasia [31]. Furthermore, the overexpressed CA19-9 modifies fibulin-3 and enhances its interaction with EGFR, suggesting that CA19-9 and EGFR play intricate roles in PDAC tumorigenesis [32].

None of the direct KRAS inhibitors have reached clinical application, despite more than three decades of intensive effort; hence, KRAS was once considered an undruggable therapeutic target [33]. This frustrating fact is partially due to the multiple alternative signalling pathways of KRAS [34–36]. Aberrantly activated RAS triggers downstream signalling by the RAF/MEK/ERK pathway, the PI3K/PDK1/AKT/mTOR pathway, RALGDS, TIAM1, and RIN1 [21]. These molecules further translocate to the nucleus and function as transcriptional modulators.

### Targeting KRAS and upstream EGFR

KRAS G12C provides a specific cysteine for drugs to bind, and thus small molecules have been designed to irreversibly bind this specific mutant target. By screening cysteine-reactive compounds, two fragments (6H05 and 2E07) were chosen as KRAS G12C-specific inhibitors [37, 38]. ARS853 was efficacious in KRAS G12C mutant cancer cells through the trapping mechanism [39], and ongoing phase I/II trials (NCT03785249 and NCT04330664) are assessing the efficacy of MRTX849, a small molecule that selectively modifies the mutant cysteine residue in KRAS G12C [40]. The relative frequency of the KRAS G12C mutation in PDAC is approximately 3% [25], suggesting that a certain subgroup of patients with PDAC may benefit from this type of treatment. In addition to small molecule inhibitors, RNA interference has been applied to target KRAS directly. Advances in endoscopic ultrasonography have assisted with the accurate placement of RNA interference molecules, such as siG12D-LODER™, into the parenchyma of patients with PDAC, and phase I/IIa trials have confirmed that this therapeutic strategy is well tolerated [41]. Engineered exosomes facilitate RNA interference efficiency as well [42] and may be applied as treatments for KRAS-mutant PDAC.

First-generation EGFR inhibitors, such as gefitinib and erlotinib, show little efficacy (median disease-free survival of patients treated with erlotinib: HR = 0.94, 95% confidence interval (CI) 0.76–1.15, *P* value = 0.26) [3, 4], partly due to the resistance caused by the non-EGFR members of the ERBB family [43, 44]. Irreversible tyrosine kinase inhibitors, such as afatinib and neratinib, have been developed to prevent the activation of the entire ERBB family. According to the results of previous clinical trials, afatinib is a more promising choice when selecting treatment for patients with KRAS-mutant lung cancer compared with gefitinib [45] or erlotinib [46], and a clinical trial of the efficacy of afatinib in patients with PDAC is ongoing (NCT02451553). Another EGFR inhibitor, nimotuzumab, improved the OS of patients with locally advanced or metastatic pancreatic cancer in a phase II trial (the median OS was 8.6 months vs 6.0 months, HR = 0.69, *P* value = 0.03), and patients with KRAS wild-type PDAC appear to benefit more from nimotuzumab than patients with KRAS mutant PDAC (the median OS was 11.6 months vs 5.6 months, *P* value = 0.03) [47]. In contrast, vandetanib failed to show efficacy (the median OS was 8.83 months vs 8.95 months, HR = 1.21, *P* value = 0.303) [48]. Another clinical trial indicated no benefit of cetuximab in the recruited patients either (the median OS was 6.3 months vs 5.9 months, HR = 1.06, *P* value = 0.23) [49]. These unsatisfactory outcomes suggest the presence of other potential resistance mechanisms that probably exist in PDAC to circumvent the inhibition of EGFR and imply that an alternative treatment strategy, i.e. the combination of EGFR inhibitors with other pharmaceuticals, may be more effective. For example, the combined inhibition of EGFR and C-RAF led to complete tumour regression in murine PDAC models and human patient-derived xenografts [50]. A phase II trial (NCT01222689) revealed modest antitumour activity following the application of erlotinib plus selumetinib to patients with locally advanced or metastatic PDAC (the median OS was 7.3 months, 95% CI 5.2–8.0 months) [51]. IGF-1R exhibits crosstalk with EGFR and mediates tumour resistance to EGFR inhibitors, and a phase II clinical trial (NCT00769483) showed that MK-0646, an IGF-1R antagonist, synergistically improved OS when applied with gemcitabine (10.4 months vs 5.7 months, *P* value = 0.02) [52]. In addition, nanoparticles (C18-EEG-GE11) have been developed to target EGFR and precisely deliver drugs to PDAC cells [53].

### Inhibiting downstream molecules of KRAS

Proteins downstream of KRAS, such as the RAF/MEK/ERK pathway or the PI3K/PDK1/AKT/mTOR pathway, have also attracted increasing interest [54, 55]. MEK is required for the viability and proliferation of tumours [23]; thus, diverse MEK inhibitors have been developed.

No significant difference was observed in the clinical trials performed to verify the efficacy of MEK inhibitors applied as a monotherapy, i.e. selumetinib and trametinib, in patients with advanced PDAC (selumetinib HR = 1.03, 80% CI 0.68–1.57, *P* value = 0.92; trametinib HR = 0.98, 95% CI 0.67–1.44, *P* value = 0.453) [56, 57]. The failures of trametinib and selumetinib appear to be due to the activation of receptor tyrosine kinases (RTKs) [58]. Accordingly, multidrug combinations of MEK inhibitors are being tested in clinical trials. High-throughput screening revealed the highest relative efficacy of AZD6244 (selumetinib) in PDAC cell lines. When applied together with AZD6244, BKM120, a PI3K inhibitor, leads to robust apoptosis in PDAC-derived organotypic models or murine models, resulting in a longer median survival (131.5 vs 71 days) [59] and indicating that the combined inhibition of MEK and PI3K may have clinical value. AKT inhibitors also produce potent synergistic effects with MEK inhibitors on PDAC [54]. Ulixertinib, an ERK inhibitor, exerts an inhibitory effect on solid tumour xenograft models [60] and appears to prevent tumour growth to a greater extent when combined with MEK inhibitors [61]. In summary, interventions that simultaneous target the two major downstream pathways of KRAS, i.e. RAF/MEK/ERK and PI3K/PDK1/AKT, represent a direction for future exploration in KRAS-mutant PDAC treatment, and clinical trials have been performed to verify the effectiveness of this strategy [62].

In addition to the simultaneous inhibition of multiple pathways, many other adjuncts to MEK inhibitors with various mechanisms have been developed. ABT-263 relieves the inhibition of BCL-XL to BIM; hence, the MEK inhibitor-induced expression of the pro-apoptotic protein BIM increases cell apoptosis and reduces the tumour volume in KRAS mutant cancer models [63]. Multiple members of the RTK/RAS/MAPK pathway have a synthetic lethal interaction with MEK, as they induce tumour resistance to MEK inhibitors by triggering an adaptive reactivation of the MAPK pathway. Therefore, the simultaneous blockade of MEK and its synthetic lethal interactors may be another strategy for KRAS mutant PDAC [58, 64–66]. SHP2 inhibition (by SHP099) and SHOC2 suppression (by gene knockout) were performed to confirm the effectiveness of this strategy in murine models. The combined application of trametinib and SHP099 or trametinib and SHOC2 knockout resulted in tumour stasis [67, 68]. In addition to the direct cytostatic effect on the tumour, MEK inhibitors also exert an inhibitory effect on several immunosuppressive immune cells, indicating potential synergy with immunotherapy. The application of GDC-0623 (cobimetinib), a MEK inhibitor, with an anti-CD40 antibody in murine models produced striking synergistic effects [69]. A strategy targeting both MEK and CDK4/6 not only delays tumour progression but also increases T-cell infiltration and tumour sensitivity to immune checkpoint inhibitors in xenograft models [70]. Interestingly, in breast cancer, the combined application of trametinib and rosiglitazone transforms cancer cells into adipocytes. This combination exploits the plasticity of cancer cells and destroys the resistance of cancer cells to conventional chemotherapy [71]. Further clinical trials assessing the efficacy of these combination therapies in PDAC will be worthwhile.

Rigosertib, an inhibitor of PI3K and PLK1, failed to improve the prognosis of patients with metastatic PDAC (OS HR = 1.24, 95% CI 0.85–1.81) [72]. In addition, paradoxically, activated AKT was observed after the inhibition of PI3K. Everolimus, an mTOR inhibitor [73], failed equally against metastatic PDAC (the median progression-free survival (PFS) was 1.8 months and the median OS was 4.5 months) [74]. Recent studies also aimed to combine PI3K inhibitors with other targeted treatments, such as MK-2206 plus selumetinib (the OS was shorter in the experimental arm, HR = 1.37, *P* value = 0.15) [75], and GDC-0941 plus ulixertinib (synergistic inhibitory activity in PDAC cell lines) [76].

### Gene fusions as promising targets in KRAS wild-type PDAC

Most patients with PDAC harbour KRAS mutations, as described above. In the small group of patients with KRAS wild-type PDAC, other mutations, such as NTRK and NRG1, initiate PDAC tumorigenesis and represent actionable targets.

Gene fusion is rare but oncogenic in KRAS wild-type cell lines [77]. The frequency of NTRK fusion and NRG1 fusion is 0.3% and 0.5%, respectively [78]. Chromosomal rearrangement of the NTRK gene family promotes the expression of tropomyosin receptor kinases with chimeric rearrangements, which are characterized by ligand-independent constitutive activation [77]. These chimeric proteins signal via the same MAPK and PI3K-AKT pathway as normal TRK proteins, and they participate in possible crosstalk with tyrosine kinases [79].

In solid tumours with NTRK gene fusions, TRK inhibitors such as larotrectinib showed significant and lasting antitumour activity, regardless of the tumour types (the overall response rate was 75%, 95% CI 61–85%) [80]. Hyperactivated chimeric TRK proteins also represent potential targets in NTRK fusion-positive PDAC. A pooled analysis of clinical trials (NCT02122913, NCT02637687, NCT02576431, NCT02097810, NCT02568267, EudraCT, and 2012-000148-88) revealed that the selective TRK inhibitors larotrectinib and entrectinib are effective against solid tumours that harbour NTRK gene fusions, including PDAC (the larotrectinib response rate was 79%, 95% CI 72–85%; and the entrectinib response rate was 57%, 95% CI 43.2–70.8%), and larotrectinib and entrectinib have received the FDA breakthrough designation of targeting NTRK fusion-positive solid tumours [81, 82]. Next-generation TRK inhibitors, such as selitrectinib and repotrectinib, are being developed to address on-target resistance [83].

NRG1 is a direct ligand of ERBB3 and ERBB4 receptors; accordingly, various NRG1 fusions initiate PDAC via the overactivation of ERBB receptor signalling pathway [84].

The ectopic ERBB signalling pathway, including constitutive activation of MEK, ERK, and PI3K, represents a potentially promising target in NRG1 fusion-initiated KRAS wild-type PDAC [85]. The anti-ERBB3 antibody GSK2849330 and pan-ERBB inhibitors afatinib and neratinib impaired cell proliferation in multiple cancer cell lines with NRG1 rearrangements. An anti-ERBB3 antibody led to tumour regression in an ovarian cancer-derived xenograft model, suggesting that the selective inhibition of ERBB3 may exert more potent antitumour effects than pan-ERBB inhibitors [86–88]. MCLA-128 (zenocutuzumab) docks on ERBB2 and blocks the binding of an NRG1 fusion protein to ERBB3. The effectiveness of MCLA-128 has been confirmed in patients with PDAC harbouring an NRG1 fusion [89]. Moreover, a phase II clinical trial of MCLA-128 in patients with solid tumours expressing an NRG1 fusion has been launched (NCT02912949).

## Tumour suppressors in PDAC and therapeutic strategies

### Dysfunctional TP53 and its reactivators

In contrast to the direct stimulation of oncogenes, tumour suppressors were originally designed to restrain tumorigenesis. Notably, p53 is a transcription factor that regulates the expression of several genes, and its biological functions include the inhibition of cell proliferation by inducing p21 expression, promoting the apoptosis of tumour cells by stimulating Bax expression, maintaining genetic stability, and inhibiting tumour vascularity [90, 91]. TP53 is the most commonly inactivated tumour suppressor in PDAC. Approximately 70% of patients with PDAC harbour alterations in the TP53 gene [23, 26].

TP53 reactivators include cys-targeting agents such as CP-31398 and APR-246, Zn^2+^ chelators such as COTI-2, and other proteins that potentially stabilize p53, help p53 refold, or inhibit the aggregation of aberrant p53 [92]. APR-246 (PRIMA-1MET) performed well in blocking the growth of haematological malignancies, prostate cancers and oesophageal adenocarcinomas [93, 94]. COTI-2 also exhibited potency in TP53-mutant squamous cell carcinoma [95]. Further studies are needed to verify whether these reactivators improve the prognosis of patients with TP53 mutant PDAC, and a clinical trial of COTI-2 is ongoing (NCT02433626). In addition to reactivation, the inhibition of murine double minute 2 (MDM2) is another emerging tactic for targeting TP53-mutant tumours. The p62-NRF2-MDM2 axis is involved in tumour progression and programming [96], and MDM2 antagonizes p53 through direct interaction or ubiquitin-dependent degradation [97]; therefore, the inhibition of MDM2 may increase the activity of p53 and restrain p53 mutant cancers [98]. Recent studies have confirmed the efficacy of MDM2 inhibitors, such as Nutlin, MA242, SP141 and MI-319, in vitro and in vivo [99–102]. However, clinical trials of MDM2 inhibitors in patients with PDAC are currently lacking.

### Dysfunctional CDKN2A and CDK4/6 inhibitors

CDKN2A is a multifunctional gene that produces p16 and p19 to arrest the cell cycle at the G1/S checkpoint through a CKD4/6-regulated mechanism [103], and the proteins bind to MDM2 to block the reduction in p53 levels [16]. Approximately 60% of patients with PDAC harbour CDKN2A mutations [23, 26], with an odds ratio of 12.33, indicating that germline mutations in CDKN2A are associated with a high risk of developing PDAC [104].

CDK4/6 is a potential target in CDKN2A-deficient tumours [105], [106]. Ribociclib and palbociclib have already shown efficacy and safety in metastatic breast cancer and liposarcoma [107, 108]. The efficacy of CDK4 inhibitors has also been confirmed in PDAC preclinical models [10–111], and related clinical trials (NCT02501902) are underway. Researchers have postulated that CDK4/6 inhibitors, which exert a limited antitumour effect as a monotherapy, show greater promise when combined with other targeted agents [112]. For instance, CDK4/6 inhibitors block the DNA repair machinery, increasing the sensitivity of PDAC cells to PARP inhibitors [113]. In addition, the combined inhibition of CDK4/6 and MEK modulates the PDAC microenvironment, increasing the sensitivity of PDAC cells to immune checkpoint blockade [70]. The application of abemaciclib and YAP1 or HuR inhibitors also exerts a synergistic inhibitory effect on PDAC cell lines [114].

### Dual role of SMAD4 in tumorigenesis and the tumour-stroma interaction

Approximately 40% of patients with PDAC harbour SMAD4 mutations [16, 23, 26]. SMAD4 mediates the pleiotropic signalling network downstream of the transforming growth factor-β (TGF-β) pathway and exerts paradoxical effects on tumorigenesis. SMAD4 prevents the tumour-promoting activity of proinflammatory cytokines and induces cell cycle arrest and apoptosis in precancerous cells. In PDAC, however, SMAD4 mutations interfere with the trimeric assembly of its C-terminal domain, which is important for its transduction activity [115], therefore preventing the normal transduction of TGF-β signals. Thus, its role switches from a suppressor to a promoter in precancerous cells [116]; moreover, TGF-β activity in mast cells induces cancer resistance to gemcitabine [117], and TGF-β suppresses the activity of normal immune cells, helping cancer cells escape from the immune system [118].

The TGF-β SMAD4 signalling pathway mediates the tumour-stroma interaction. PDAC has two distinct epithelial-mesenchymal transformation (EMT) subtypes, the complete EMT and partial EMT, and the latter is speculated to result in an increased metastasis rate via the formation of clusters of circulating tumour cells [119]. Cancer-associated fibroblasts secreting TGF-β may induce the partial EMT and switch PDAC proliferation phenotypes, contributing to PDAC heterogeneity [120]. PDAC with an impaired TGF-β-SMAD4 signalling pathway per se may modulate the fibrotic response and mechanophenotype [121], indicating that molecular alterations in tumours not only control PDAC progression but also reprogram the metabolic phenotypes of cells in the TME. Heterozygous mutation of SMAD4 attenuates the metastatic potential of PDAC cells while increasing their proliferation. Reportedly, SMAD4 is also correlated with glucose transporter expression and matricellular fibrosis. Clinical studies have confirmed that SMAD4 inactivation is associated with a poor prognosis [122, 123].

Because of the dual roles of SMAD4 in cancer cells, agents have been designed to inhibit rather than activate TGF-β in SMAD4-deficient tumours [124, 125]. Galunisertib, a TGF-β inhibitor, showed efficacy in a preclinical investigation [126]. Phase I/II trials showed that the combined application of galunisertib and gemcitabine prolonged OS (estimated HR = 0.796) [127, 128].

### Roles of SMAD4 and related molecules in PDAC subtyping

The RUNX3 expression level is strongly correlated with the SMAD4 status. Accordingly, RUNX3 also functions as both a tumour suppressor and promoter in PDAC and regulates the balance between cancer cell proliferation and dissemination. RUNX3 combined with DPC4 helps distinguish PDAC subtypes and enables more precise clinical decisions [129]. In SMAD4-negative PDAC, PGK1 is selected as the decisive gene to determine the PDAC metabolic phenotype and balance metastasis and proliferation. Nuclear PGK1 determines the metastatic potential of PDAC cells, thus helping to predict metastatic patterns of PDAC cells and providing guidance for precise therapy [130].

## Role of epigenetics in PDAC

In a recent genomic analysis, the molecular features of PDAC were reclassified into four subtypes, among which the squamous subtype correlated with hyper-methylation and concordant downregulation of genes that regulate endodermal cell differentiation [131]. Histone methylation both induces and represses gene expression. Based on accumulating evidence, alterations in histone methylation modulate multiple biological processes. Polycomb repressive complex 2-mediated histone H3 lysine 27 trimethylation (H3K27me3) is correlated with transcriptional repression [132]. Dimethylases such as KDM6A regulate endoderm differentiation by removing the aforementioned H3K27me3 methylation mark. During endoderm differentiation, KDM6A upregulates WNT3 expression in the early stage, while increasing DKK1 expression in the late stage. Therefore, KDM6A exerts dual effects on the WNT pathway and plays a cell identity-safeguarding role [132].

The KMT2C(MLL3)-KDM6A(UTX)-PRC2 regulatory axis modulates the expression of various downstream tumour suppressor genes, and thus the inactivation of KDM6A results in the activation of super-enhancers and contributes to the squamous subtype of PDAC in females [133]. UTY compensates for the KDM6A deficiency in males, and simultaneous inactivation of KDM6A and UTY will also induce the formation of the squamous subtype of PDAC. Accordingly, resetting the balance of this axis represents a new approach for PDAC therapy.

In vitro and in vivo trials have confirmed that GSK126, an EZH2 inhibitor, rescues the expression of downregulated genes in MLL3 knockdown cells, indicating that EZH2 represents a potential therapeutic target for MLL3 mutant cancers [133]. A deficiency in KDM6A also confers sensitivity to bromodomain and extra-terminal domain (BET) inhibitors such as JQ1 in PDAC. BET inhibitors restore the cell identity by reducing the activity of the MYC pathway and decreasing p63 levels [134]. Combined inhibition of BET and histone deacetylases exerted synergistic effects on reducing cell viability [131]. Future investigations of therapeutics targeting genes that regulate epigenetics are intriguing.

## DNA damage repair and synthetic lethality

**Fig 2:**
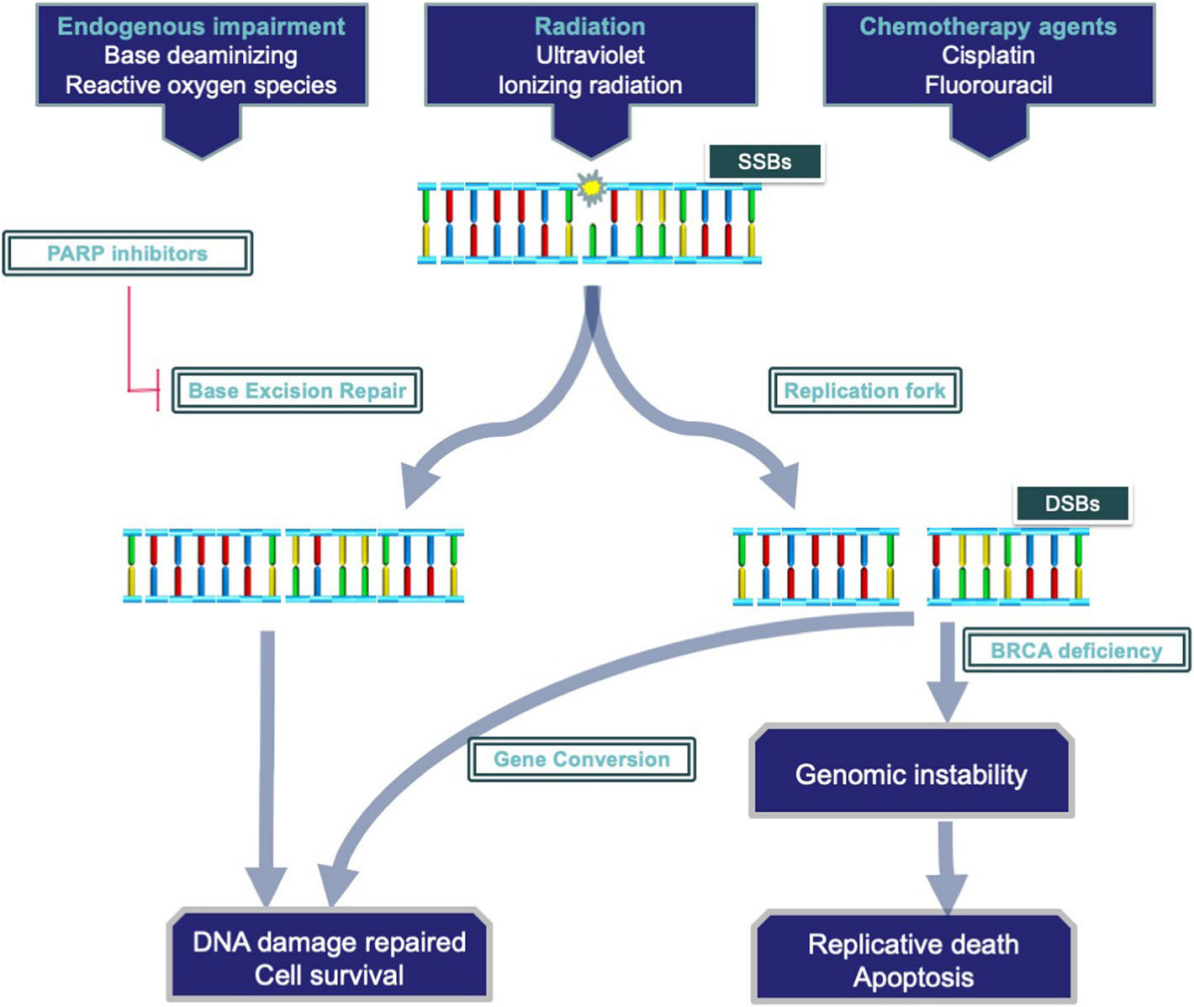
Various factors could cause DNA single-strand breaks (SSBs). SSBs are repaired by poly (ADP-ribose) polymerase (PARP) through the base excision repair (BER) mechanism. Therefore, the application of PARP inhibitors will enable BER and cause many SSBs. These lesions will transfer to DNA double-strand breaks (DSBs) during cell proliferation. DSBs are repaired by BRCA through the gene conversion (GC) pathway in normal cells. However, in BRCA-loss cancer cells, DSBs cannot be repaired and will lead to fatal genomic instability

Cells with DNA damage may ultimately die or acquire oncogenic potential; thus, multiple mechanisms have been established to prevent such lethal or oncogenic DNA lesions [135]. BRCA is implicated in assisting the recombinase function of RAD51 in the gene conversion (GC) pathway to repair DNA double-strand breaks (DSBs) [136–138]. PARP-1 is involved in the base excision repair (BER) pathway to repair DNA single-strand breaks (SSBs), and thus its inhibition will lead to a failure to repair these DNA lesions, which subsequently results in DSBs when a DNA replication fork is encountered [139]. Thus, the application of PARP inhibitors to BRCA-deficient cells will cause significant lethal effects (Fig. 2). PDAC has been divided into four subtypes according to the structural rearrangements, and the unstable subtype is most sensitive to DNA-damaging agents [140].

Synthetic lethality was discovered in fruit flies and yeast decades ago [141, 142]. If two genes have collaborative biological functions, an organism in which either gene alone is perturbed is viable, whereas the simultaneous perturbation of both genes causes a synthetic lethal effect. Therefore, the identification of deletion mutations in genes that are implicated in a certain synthetic lethality in tumours and then inhibiting their counterparts is a feasible treatment to selectively target tumour cells [143].

BRCA is an ideal synthetic lethal target. BRCA-deficient cells repair DSBs through error-prone pathways that contribute to genomic instability, resulting in cell death or oncogenesis [144–146]. Individuals with BRCA germline mutations have a remarkably increased risk of pancreatic cancers [137], breast cancer, and ovarian cancer [147]. The frequency of BRCA mutations is approximately 5.9–7.2% in PDAC [148–150], suggesting that a certain group of patients with PDAC may benefit from PARP inhibitors.

PARP inhibitors have already shown notable efficacy against other refractory BRCA mutant solid tumours [151–154]. Olaparib, a PARP inhibitor, was efficacious in a single-arm phase II trial [152]. More recently, a prospective phase III trial (the POLO trial, Pancreas Cancer Olaparib Ongoing, NCT02184195) was performed to evaluate the efficacy of olaparib in patients with BRCA mutant metastatic PDAC [155]. The PFS was apparently increased in the olaparib group (7.4 months versus 3.8 months, HR = 0.53, *P* value = 0.004). Significant differences in other indicators, including OS, second PFS and the objective response rate, were not observed between the groups. The POLO trial also verified the safety of olaparib [153, 156].

Considering the poor prognosis of patients with PDAC, improving the OS may be more meaningful than improving PFS; nevertheless, the prolonged PFS suggested that a subgroup of patients with metastatic PDAC carrying BRCA mutations may benefit from olaparib maintenance therapy [157]. PARP inhibitors require more rigorously designed trials to confirm their efficacy against BRCA mutant PDAC.

Synthetic lethality exploits the intrinsic deficiency of tumours, exhibits high selective toxicity and offers a wide therapeutic window. For example, SMARCA, MYC and ARID also exert vital biological functions; therefore, treatments exploiting their deficiency in tumour cells will provide a new direction for precisely targeted therapy in certain PDAC subgroups.

## The immunosuppressive microenvironment and immunotherapy in patients with PDAC

The human immune system recognizes and kills incipient tumour cells. Correspondingly, a critical point in tumour formation is evading immune surveillance [158]. Cancer cells escape immune destruction through multiple approaches, including tumour-associated antigen modulation, the acquisition of low immunogenicity, and induction of an immunosuppressive TME. According to a transcriptomics analysis, a proinflammatory immune component already exists in low-grade preneoplastic lesions [19]. During PDAC progression, the TME transforms into an immune-evading phenotype, and various types of immune cells are induced to become anergic or immunosuppressive [121, 159–161]. The major barrier of immunotherapy in PDAC has been the fibrotic stroma, which forms a physical barrier to prevent lymphocyte infiltration [162]. As our understanding of oncology and immunology improves, immunotherapy is predicted to remove these tumour immune-resistant mechanisms and restore the normal antitumour immune response.

### Chimeric antigen receptor T cells (CAR-T)

CAR-T is a hotspot of immunotherapy. The autologous T cells of patients are isolated and reprogrammed to precisely target tumour-associated antigens [163]. CAR-T has already proven to be effective against haematological neoplasms [164], and the FDA has approved Kymriah and Yescarta, two CAR-T drugs targeting CD19-expressing cancer cells, for clinical application [165, 166]. In addition to CD19, other characteristic surface biomarkers of solid tumours also have the potential to be designed as CAR-T therapeutic targets, as shown in Table 2. For example, the diverse tumour-specific glycosylated antigens provide a roadmap for CAR-T targets [167, 168]. CAR-T targeting the abnormal O-glycosylation site, i.e. the Tn and STn antigens on MUC1, has already been shown to inhibit the growth of PDAC cell lines [169] and control PDAC xenograft growth in murine models [170]. The combination of CEA-CAR-T with rhIL-12 exerted significant antitumour effects in vitro and in vivo [171], and a phase II/III trial (NCT04037241) to evaluate the efficacy of CEA-CAR-T is recruiting patients. CD133 is a marker of cancer stem cells and is related to tumour metastasis and recurrence; a phase I trial (NCT02541370) confirmed the safety of CAR-T-133 in patients with advanced metastatic malignancies [172]. Mesothelin (MSLN) is implicated in tumour invasion and is widely overexpressed in solid tumours, including PDAC [173]. The targeting of mesothelin by CAR-T controls the metabolic active volume in murine models [174], and a phase I trial (NCT02159716) suggested that MSLN CAR-T is safe in patients with solid tumours, including PDAC [175]. Moreover, dual-receptor CAR-modified T cells that simultaneously recognize CEA and MSLN were designed to attenuate the “on-target, off-tumour” toxicity [176]. The appealing KRAS protein is also involved in the exploration of CAR-T; experiments using CAR-T targeting mutant KRAS G12D suggested that the loss of heterozygosity at the HLA may reduce the efficacy of immunotherapy, and a phase II trial (NCT01174121) of this CAR-T is ongoing [177]. HER2/ERBB2 is a transmembrane protein that induces tumour initiation and progression; therefore, HER2 potentially represents an ideal target, and the safety of CAR-T-HER2 has been confirmed in a phase I trial (NCT01935843) [178]. In addition, a study used switchable CAR-T targeting HER2 to increase its efficacy and reduce its toxicity [179]. Programmed cell death protein-1 (PD-1) is a famous immune checkpoint receptor that is involved in tumour immune evasion. In addition to small molecule inhibitors, chPD1 T cells have been designed to target PD1 precisely, and a preclinical study observed protective antitumour responses of chPD1 T cells in multiple models of solid tumours [180]. B7-H3 overexpressed on the PDAC cell surface is another attractive target, xenograft PDAC models certified the effectiveness of CAR-T targeting B7-H3, and 4-1BB co-stimulation enhanced this antitumour activity [181].

**Table 2:** Tumour-associated antigens and corresponding CAR-Ts, ADCs or BiTEs

Tumour-associated antigens (targets)	Biological function	Agent	Study phase	Research tumour type	Reference
Tn-MUC1 Sialyl-Tn-MUC1	Alter cancer cell adhesion and motility	5E5 CAR T	Mouse Model	Leukemia, PDAC, Breast cancer	2016, Immunity
B7-H3	T cell co-stimulatory molecule	B7-H3. CAR T	Patient derived xenograft	PDAC, Ovarian cancer, Neuroblastoma	2019, Cancer Cell
Mesothelin	Tumour local invasion and metastasis	MSLN CARs	Phase I	Mesothelioma, Ovarian carcinoma, PDAC	NCT02159716
		Anetumab ravtansine	Phase I	Mesothelioma, Ovarian carcinoma, PDAC, etc	NCT03102320
CEA	Tumour surface biomarker	CEA-CAR-T	Mouse models	Colorectal cancer, Gastric cancer, PDAC	2019, Cancer Medicine
			Phase II/III	PDAC	NCT04037241
Mesothelin & CEA		dCAR-T	Cell models	PDAC	2018, Journal of Hematology and Oncology
KRAS G12D HLA-C*08:02	Tumour formation and progression	CTL targeting KRAS G12D	Phase II	Metastatic cancers (Colorectal cancer, Glioblastoma, PDAC, Ovarian cancer, Breast cancer)	2016, New England Journal of Medicine NCT01174121
HER2/ERBB2	Tumorigenesis and tumour proliferation	Switchable CAR T against HER2	Xenograft model	PDAC	2019, Gut
		CART-HER2	Phase I	Biliary tract cancer, PDAC	NCT01935843
		DS-8201a	Phase I	Solid tumors	2016, Clinical Cancer Research
CD133	Tumour stem cells marker	CAR T-133	Phase I	Hepatocellular carcinoma, Colorectal carcinoma, PDAC	NCT02541370
PD-1	Immune checkpoint	chPD1 T cells	Mouse model	Solid tumors (melanoma, renal cancer, liver cancer, PDAC, etc.)	2020, Immunology
MUC16	Tumour surface biomarker	DMUC5754A	Phase I	Ovarian cancer, PDAC	NCT01335958
Guanylyl cyclase C	Membrane receptor	MLN0624	Phase II	PDAC	NCT02202785
Glypican-1	Cell surface proteoglycan	GPC-1-ADC	Patient derived xenograft	PDAC	2020, British Journal of Cancer
EpCAM	Cell adhesion	MT110	Phase I	Colorectal cancer, Ovarian cancer, Gastric cancer, Lung cancer, Prostate cancer	NCT00635596

*PDAC* pancreatic ductal adenocarcinoma; *CAR-T* chimeric antigen receptor T cells; *ADC* antibody-drug conjugate; *BiTE* bispecific T-cell engager; *MSLN* Mesothelin; *CTL* cytotoxic T lymphocytes; *PD-1* programmed death-1 receptor

### Antibody-drug conjugates and bispecific T-cell engagers

In addition to CAR-T, antibody-drug conjugates (ADC) and bispecific T-cell engagers (BiTE) are also designed to confer selective toxicity to PDAC cells. ADC combine antibodies against tumour-specific antigens with cytotoxic agents; hence, cell toxins are able to precisely target cancer cells.

The most common cell toxins are microtubule-disrupting agents. For example, DMUC5754A conjugates an anti-MUC16 antibody to monomethyl auristatin E (MMAE); however, it was ineffective at treating patients with PDAC in phase I trial [182]. MLN0624 conjugates anti-guanylyl cyclase C to MMAE, and it is reported to have a limited benefit for patients with PDAC [183]. A glypican-1 antibody has been conjugated to monomethyl auristatin F (MMAF) and significantly inhibits the growth of xenografts derived from patients with PDAC [184]. Anetumab ravtansine conjugates an anti-mesothelin antibody to the tubulin inhibitor DM4, and it exhibited great tolerance in a phase I trial and warrants future investigation [185].

In addition to cytoskeleton-disrupting agents, other drugs have also been conjugated to antibodies, such as DS-8201a, which conjugates a topoisomerase I inhibitor with HER-2 antibodies. A phase I trial supported the use of DS-8201a as a potentially promising treatment [186].

BiTEs simultaneously bind tumour-associated antigens and the CD3 epitope on the T cell surface, forming an immune synapse and resulting in the targeted lysis of tumour cells [187]. For example, MT110 (solitomab) links EpCAM with CD3 and redirects T cells to selectively kill PDAC cells [188]. However, a phase I trial revealed adverse events of solitomab and prevented dose escalation to therapeutic levels [189].

### Immune checkpoint inhibitors

Immune checkpoint inhibitors, such as ipilimumab and nivolumab, also show potential in antagonising tumours [190]. An increasing number of trials have been designed to combine PD-1 or programmed cell death 1 ligand 1 (PD-L1) inhibitors with other treatments [191]. However, only a subgroup of tumours are sensitive to immune checkpoint blockade; thus, indicators are required to guide the treatment more efficiently [192]. The tumour mutational burden exhibits a strong linear correlation with the objective response rate to PD-1 inhibition. PDAC with a low number of genomic mutations is more resistant to PD-1 inhibitors than PDAC with a high number of genomic mutations [193]. A high degree of microsatellite instability (MSI-H) results in a high tumour mutational burden [194]. Therefore, mismatch repair deficiency (dMMR) and subsequent MSI-H are good predictors of the efficacy of PD-1 or PD-L1 inhibitors [195]. The latest phase II KEYNOTE-158 trial revealed a benefit of PD-L1 inhibitors in combination with pembrolizumab in patients with MSI-H/dMMR cancers (the objective response rate in the pancreatic cancer subgroup was 18.2%, 95% CI 5.2–40.3%) [196]. Approximately 1% of patients with PDAC exhibit dMMR/MSI-H; therefore, the clinical value of applying PD-1 or PD-L1 antibodies in PDAC is limited.

## Conclusions and prospects

Targeted therapy aims to kill cancer cells with high selectivity, and thus its key goals are recognizing certain patient subgroups and identifying targets that are specific to tumours. Advances in NGS have facilitated the PDAC diagnosis and contribute to the categorization of PDAC into different subtypes. In PDAC, the four major driver genes and their pleiotropic signalling networks provide a framework for exploring ideal targets. Furthermore, low-frequency mutated genes with vital biological functions help discriminate certain PDAC subtypes and guide future precision oncology (Table 3).

**Table 3:** Recent major and pivotal clinical trials for targeted therapy in PDAC

Agent	Therapeutic mechanism	Target	Study phase	Numbers of patients (with PDAC)	Efficacy	Clinical trial	Reference
Erlotinib	Tyrosine kinase inhibition	EGFR	Phase III	436	DFS and OS not improved	CONKO-005	DRKS00000247
			Phase III	449	OS not improved	LAP07	NCT00634725
Vandetanib		EGFR, RET, VEGFR2	Phase II	142	OS not improved		EudraCT2007-004299-38, ISRCTN96297434
Nimotuzumab	Monoclonal antibody	EGFR	Phase IIb	186	Longer OS in KRAS^WT^, HR = 0.69		EudraCT2007-000338-38, OSAG101-PCS07, NCT00561990
MK-0646		IGF-1R	Phase II	75	OS improved		NCT00769483
MCLA-128 (Zenocutuzumab)		ERBB3	Phase II	recruiting			NCT02912949
Selumetinib and MK-2206	Oncogenic pathway inhibition	PI3K and MEK	Phase II	137	PFS and OS not improved	SWOG S1115	NCT01658943
Olaparib	Synthetic lethality	PARP	Phase III	164	Longer PFS, HR = 0.53	POLO trial	NCT02184195
Pembrolizumab	Immune checkpoint blockade	PD-1	Phase Ib	24	ORR = 0	KEYNOTE-028	NCT02054806
			Phase II	22	ORR = 18.2	KEYNOTE-158	NCT02628067
CAR T	Target tumour-associated antigens	HER2	Phase I	2	SD = 2		NCT01935843
		Mesothelin	Phase I	5	SD = 3, PD = 2		NCT02159716
		CD133	Phase I	7	PR = 2, SD = 3, PD = 2		NCT02541370

*PDAC* pancreatic ductal adenocarcinoma; *DFS* disease-free survival; *OS* overall survival; *KRAS*^*WT*^ KRAS wild-type; *PFS* progression-free survival; *HR* hazard ratio; *ORR* objective response rate; *PD-1* programmed death-1 receptor; *SD* stable disease; *PR* partial response; *PD* progressive disease

KRAS is undoubtedly an attractive target in PDAC. Specific KRAS mutant residues, such as the cysteine residue in KRAS G12C, may be modified by small-molecule compounds such as MRTX849 and ARS853. Furthermore, RNA interference and exosomes are being developed to directly target KRAS.

KRAS-related molecules and pathways are also research hotspots. Researchers have attempted to target related molecules, such as EGFR, MEK and PI3K. With the exception of erlotinib and nimotuzumab, EGFR inhibitors all failed in clinical trials, indicating the presence of underlying mechanisms in PDAC to resist EGFR inhibitors. Trials aimed at evaluating the efficacy of pan-ERBB inhibitors, such as afatinib, in PDAC are underway. In addition, the combination of EGFR inhibitors with drugs targeting multiple molecules may be a more promising approach. Monotherapy with MEK inhibitors, such as selumetinib and trametinib, did not improve the prognosis of patients with PDAC in clinical trials. An emerging trend is to combine MEK inhibitors with other agents, such as ABT-263, BKM120, SHP099, and ulixertinib. MEK also participates in modulating the TME and regulating the EMT in PDAC, and thus can be utilized in various therapeutic strategies. Based on the aforementioned research outcomes, future studies targeting KRAS-related pathways may focus on interventions targeting multiple dysregulated molecules and elucidating the resistance mechanisms.

Gene fusions, such as NRG1 and NTRK, are important oncogenes in KRAS wild-type PDAC, and hyperactivated chimeric TRK proteins and the ectopic ERBB signalling pathway represent potential therapeutic targets in patients with PDAC presenting aberrant NTRK and NRG1 function, respectively.

Mutations in tumour suppressors, mainly alterations in TP53, SMAD4 and CDKN2A, also contribute to tumorigenesis in PDAC. These molecules are implicated in sophisticated molecular networks and play intricate roles in tumour initiation and progression; thus, many possible strategies are potentially useful to target these proteins. Agents have been developed to directly reactivate tumour suppressors or target-related molecules, such as MDM2, CDK4/6 and TGF-β. Their success in other tumours are expected to be repeated in PDAC, and their preclinical achievements in PDAC are also expected to transfer to clinical applications. Newly developed therapeutic strategies, such as gene editing and synthetic lethality, are conceivable dark horses that are potentially useful for targeting these intrinsically deficient cancer cells, but further trials are required to confirm their potential.

Epigenetic genes regulate chromatin modulation, and therefore control the expression of other genes, suggesting that epigenetic genes are potential therapeutic targets. BET inhibitors and EZH2 inhibitors were designed to rescue the dysregulated KMT2C(MLL3)-KDM6A(UTX)-PRC2 regulatory axis and achieved preliminary success in preclinical models. Cells that harbour a deficiency in the DNA repair machinery have a higher risk of becoming cancerous. Correspondingly, PARP inhibitors are designed to selectively kill BRCA mutant cancer cells. Recently, partial efficacy of olaparib was confirmed in clinical trials. Although the results were not ideal, the associated controversies have prompted more investigations to achieve synthetic lethality in PDAC.

Immunotherapy remains a future breakthrough in the treatment of PDAC. A growing number of CAR-T targets have been identified, such as mesothelin, CEA, CD133, Tn/STn, B7-H3, KRAS G12D, PD-1 and HER2. ADC and BiTEs have also been developed to target PDAC cells precisely. The positive results of these treatments in preclinical studies suggest promising applications, and many of these molecules are being investigated in ongoing clinical trials. In addition to CAR-T therapy, immune checkpoint blockade, such as PD-1 or PD-L1 antibodies, also shows potential. The tumour mutational burden has been suggested to be related to the objective response rate to PD-1 inhibitors, and pancreatic cancer with a low number of genomic mutations is generally resistant to PD-1 or PD-L1 inhibitors. Notably, dMMR/MSI-H may predict the efficacy of PD-1 or PD-L1 inhibitors, but only 1% of patients with PDAC exhibit dMMR/MSI-H. Nonetheless, the rapid development of immunotherapy is still anticipated.

Targeted therapy will definitely provide diverse therapeutic strategies for PDAC and improve its poor prognosis. The high frequency of mutations in the four major driver genes indicates their great importance; therefore, future directions of precise oncology in PDAC will still focus on the four major driver genes and related signalling pathways. Low-frequency mutant genes will also help to distinguish curable subgroups of patients with PDAC who harbour mutations in specific targets, and they will thus be treated more accurately. Hopefully, PDAC will be completely treatable using these approaches.

## Data Availability

Not applicable.

## Abbreviations

ADC: Antibody-drug conjugate
BER: Base excision repair
BET: Bromodomain and extra-terminal
BiTE: Bispecific T-cell engager
CI: Confidence interval
CAR-T: Chimeric antigen receptor T cell
dMMR: Mismatch repair deficiency
DSB: DNA double-strand break
EGFR: Epidermal growth factor receptor
GC: Gene conversion
H3K27me3: histone H3 lysine 27 trimethylation
HR: Hazard ratio
MDM2: Murine double minute 2
MMAE: Monomethyl auristatin E
MMAF: Monomethyl auristatin F
MSI-H: Microsatellite instability-high
MSLN: Mesothelin
OS: Overall survival
PD-1: Programmed cell death protein 1
PD-L1: Programmed cell death 1 ligand 1
PDAC: Pancreatic ductal adenocarcinoma
PFS: Progression-free survival
POLO: Pancreas Cancer Olaparib Ongoing trial
RTK: Receptor tyrosine kinases
SSB: DNA single-strand break
TGF-β: Transforming growth factor-β
TME: Tumour microenvironment
